# Male Infertility: From Genes to Genomes 2022

**DOI:** 10.3390/genes14050959

**Published:** 2023-04-23

**Authors:** Maria-Anna Kyrgiafini, Zissis Mamuris

**Affiliations:** Laboratory of Genetics, Comparative and Evolutionary Biology, Department of Biochemistry and Biotechnology, University of Thessaly, Viopolis, Mezourlo, 41500 Larissa, Greece

Infertility is defined as the inability to conceive after at least 12 months of regular, unprotected sexual intercourse and it is considered an alarming global health issue. More importantly, it is a unique medical condition, since it involves a couple rather than a single individual. Especially today, a couple’s journey to parenthood is not always as clear-cut as it is made out to be. Male infertility affects 7% of the global male population and it is estimated that the male factor contributes to approximately half of all cases of infertility [1]. Even though it is clear that genetic causes play a role in male infertility, there is a distinct lack of diagnostically relevant genes, and at least 40% of all cases are classified as idiopathic [1,2,3]. Furthermore, declining semen quality over the past 50 years suggests the influence of changing lifestyles and exposure to environmental and toxic components, although the exact mechanisms are not yet clearly understood [4]. However, in the modern era of genomics, next-generation sequencing (NGS) technologies, such as whole-exome and -genome sequencing, offer the opportunity to simultaneously study numerous genes and identify new biomarkers.

In this context, this Special Issue, titled “Male Infertility: From Genes to Genomes 2022”, focuses on research advances in male infertility with a particular emphasis on the identification of new genetic variants and genes that contribute to this phenotype. The submissions, comprising two reviews and six original research articles, cover many different aspects of the complex phenotype of male infertility. 

Both reviews provide excellent overviews of the state of the art regarding male infertility. Amor and Hammadeh (2022) [5] performed a systematic review of the role of mitochondrial variants in male infertility. The authors highlight that mutations in mtDNA have recently been associated with many diseases, such as diabetes mellitus type 2, various cancers, etc., but their association with male infertility remains largely unexplored. In their review, they report many variants that mainly affect energy production and the function of mitochondrial oxidative phosphorylation (OxPhos) machinery leading to reduced sperm motility. Furthermore, they draw attention to examples of paternal inheritance of mtDNA, a phenomenon that can have serious implications in cases of non-normozoospermic men giving birth to offspring through assistive reproductive technology. Kaltsas et al. (2023) [6] provide another valuable contribution to our Special Issue, addressing the question of how the increasing paternal age affects fertility or the health of offspring, as in recent years, increasing anomalies have been reported in middle-aged couples during pregnancy. The authors review the existing evidence and show an association between paternal age and decreased sperm quality, as well as testicular function. It also seems that fertility levels start to drop after the age of 35; however, the number of these studies is limited. Sperm DNA damage, telomere length, and chromosome aberrations are some of the sperm genetic and epigenetic changes that are associated with increased paternal age and may cause the decline in sperm quality. All of the above can even affect the success rate of assisted reproductive technologies or lead to miscarriage. Finally, children with older fathers are at higher risk for health defects and diseases such as autism and schizophrenia. Taking these into consideration, the authors conclude that there is a growing need for guidance and consultation for couples during their reproductive years.

Six original submissions also provide significant insights into various aspects of male infertility. Two of these articles are studies of human subjects. Punjabi et al. (2023) [7] performed an interesting study in fertile (F), subfertile normozoospermic (SN), and subfertile non-normozoospermic males (SN-N) to evaluate genome instability among the three groups with various assays (sperm DNA fragmentation (SDF), sperm chromatin maturity, and sperm aneuploidy). Surprisingly, normozoospermic men with semen parameters above the WHO lower reference values may still carry spermatozoa with DNA damage, suggesting that the lower reference limits are not sensitive enough to predict the chances of conception. More specifically, in this study, no statistically significant difference was observed among the three groups for SDF, and the frequency of aneuploidy sperm between the SN and SN-N groups was not significantly different. When compared to the F group, chromatin decondensation was significantly reduced and hyperstability was significantly increased in the SN group. These intriguing findings bring to the forefront the need for continuous research aimed at improving the accuracy and sensitivity of semen analysis or at the identification of additional parameters that could be included in future WHO guidelines to identify men with fertility problems with greater sensitivity. Therefore, it is suggested that genome instability may be an independent characteristic of sperm quality, detecting problems that sperm analysis alone does not detect. Kyrgiafini et al. (2022) [8] used a whole-genome sequencing approach to analyze the genomes of men diagnosed with teratozoospermia, and through a comparison with the genomes of normozoospermic men they identified several genetic variants and genes that may play a role in the development of this condition. More specifically, after variant prioritization and bioinformatics analysis they detected high-impact variants in approximately sixty genes. Many of these genes have been previously associated with male infertility, yet others are related for the first time to teratozoospermia, paving the way for future studies. Notably, pathway enrichment analysis indicates that the extracellular matrix (ECM) may be involved in the development of teratozoospermia. Although many studies have previously reported the important role of ECM in the spermatogenesis process, there are no studies directly associating the extracellular matrix with teratozoospermia. Finally, this study also highlights the importance of using whole-genome sequencing to identify genetic variants that may be missed by other approaches.

Furthermore, many experimental studies using mouse models have been included in this Special Issue. Shi et al. (2021) [9] performed a yeast two-hybrid screen to shed light on the molecular mechanisms of cAMP-dependent protein kinase (PKA) signaling, which is extremely important for mammalian spermatogenesis. More specifically, the researchers aimed to identify proteins that interact with PKA-Riα (PKA regulatory subunit I α) during mouse spermiogenesis. They found that PKA-Riα interacts with several proteins involved in various cellular processes, such as cytoskeletal organization, protein synthesis and protein folding, mRNA processing, etc. Many of the proteins identified have also been reported in previous studies to be important for the development of sperm or to exert a testis-specific expression pattern. Notably, metabolic enzymes were also found to interact with PKA-Riα, though their functional role in spermiogenesis is unknown. The authors suggest that these interactions may play a crucial role in sperm development and maturation. These findings provide insights into the molecular mechanisms underlying spermiogenesis and highlight potential targets for future research in the field of male infertility. Mouse models were also used in the study performed by Bang et al. (2022) [10] to investigate the role of hyaluronidase 6 in male fertility. Though glycosylphosphatidylinositol-anchored sperm hyaluronidases have long been thought to contribute to sperm penetration through the cumulus–oocyte complex (COC), the role played by sperm hyaluronidases in mammalian fertilization is still up for dispute. A characteristic example is that HYAL5 and HYAL7 are not independently essential for fertilization, but according to a previous study double-knockout mice deficient in both *Hyal7* and *Hyal5* were 90% less fertile in comparison with WT mice and had a fertilization rate of less than 5% with in vitro fertilization (IVF) [11]. In this study, researchers focused on the role of HYAL6, which is found in the sperm head, indicating a role in fertilization. The authors generated HYAL6 KO mouse models using CRISPR/Cas9 and, contrary to expectations, it was observed that HYAL6 KO male mice had normal fertility and sperm characteristics. The COC dispersal was not affected, and after an IVF assay used to assess the interaction between HYAL6 KO mouse sperm and egg no difference in fertility was observed between KO and WT mice. All these findings prove that HYAL6 is not required for fertilization, and it has no hyaluronidase activity. Furthermore, in efforts to improve anticancer therapy and minimize long-term toxicity, the study of Nagahori et al. (2022) [12] evaluates the effect of single irradiation and re-irradiation on specific mRNA transcripts of mouse testes. Radiotherapy is a commonly used cancer treatment and there is an extensive body of literature on its effects on gonadal function. Though it is known that irradiation can lead to infertility, the relationship between the negative consequences and accumulated doses of irradiation from repeated low-dose radiation exposure, as in the case of re-irradiation, in comparison with single irradiation, is not well understood. In their study, Nagahori et al. (2022) [12] found that re-irradiation caused noticeably reduced testicular weight, disrupted spermatogenesis, and a considerable decline in mRNA species associated with germ cell differentiation. Furthermore, nearly half of the Sertoli-cell-specific mRNA species that decreased upon irradiation exhibited significant variations between single- and re-irradiation, indicating that different mechanisms that require further investigation may be involved in long-term aspermatogenesis after single- and re-irradiated treatment.

Finally, Dewaele et al. (2022) [13] investigate the role of estrogens in male infertility using a rabbit model. Although estrogens are considered “female sex hormones”, they are also produced in the testes of mammals, and more specifically, cytochrome P450 aromatase catalyzes the irreversible conversion of androgens to estrogens. Additionally, it is known that estrogens exert their function through nuclear receptors ESR1 and ESR2. At first, Dewaele et al. (2022) [13] studied the expression of *CYP19A1*, *ESR1*, and *ESR2*. Their analysis revealed that testicular estrogens are produced in the seminiferous tubules (mainly by meiotic germ cells), whereas the expression of both ESR1 and ESR2 was detected in round spermatids, suggesting that estrogens may have a role in post-meiotic germ cells. Then, they used CRISPR/Cas9 CYP19A1^−/−^ genetically modified rabbits to examine the effect of testicular estrogen deprivation on testes and sperm production. Notably, the rabbits exerted impaired spermatogenesis, which led to lower sperm count, increased morphological abnormalities, and decreased spermatozoa motility. The authors conclude that the phenotype of CYP19A1^−/−^ rabbits resembles the rare cases of aromatase mutations observed in humans, suggesting that the rabbit can be a very useful biomedical model for the study of male infertility.

In summary, the collection of articles included in this Special Issue, “Male Infertility: From Genes to Genomes 2022”, provides novel insights for all researchers and healthcare professionals engaged in the field of male infertility. We also hope that these findings will inspire researchers and pave the way for many more studies that will provide answers to many biological questions associated with male fertility.
